# Conception of a mobile health application targeting early postoperative physiotherapeutic care after total knee replacement, a qualitative study

**DOI:** 10.3389/fsurg.2024.1283202

**Published:** 2025-01-27

**Authors:** Hassan Tarek Hakam, Nikolai Ramadanov, Awda Garzuzi, Mikhail Salzmann, Hannes Hofmann, Felix Muehlensiepen, Roland Becker, Robert Prill

**Affiliations:** ^1^ Center of Orthopedics and Traumatology, Brandenburg Medical School (MHB-Fontane), University Clinic of Brandenburg, Brandenburg, Germany; ^2^ Faculty of Health Sciences, University Clinic of Brandenburg, Brandenburg, Germany; ^3^ Center of Evidence Based Practice in Brandenburg (EBB), A JBI Affiliated Group, Brandenburg, Germany; ^4^ Self employed, Berlin, Germany; ^5^ Center for Health Services Research, Faculty for Health Sciences, University Clinic of Brandenburg, Berlin, Germany

**Keywords:** mobile health application, physical therapy, rehabilitation, total knee arthroplasty, telerehabilitation

## Abstract

**Introduction:**

Mobile applications targeting physiotherapeutic care after total joint replacements are increasing in popularity among end-users. However, these applications were primarily conceived out of financial interest and lack an evidence-based programs tailored to the specific needs of the target population. The primary objective of this study is to describe the conception of an evidence-based mobile health application that targets early postoperative physiotherapeutic care after total knee replacement (TKR).

**Methods:**

A literature search of eHealth applications targeting physical therapy after TKR was carried out. Articles were then screened and suggestions as well as recommendations were extracted to inform the design of a new application. The beta version of the application was then passed onto experts for evaluation. Final changes were then undertaken to account for the expert's opinions.

**Results:**

Several reviews with recommendations for the design of applications targeting patients after total joint replacement were identified. Primarily, mobile applications targeting rehabilitative care after TKR need to be tailored to the needs of the elderly population. Additionally, no unified rehabilitative physiotherapeutic (PT) program was found reflecting a discrepancy regarding what exercises are most useful. A comparison of different programs yielded no significant difference favoring one single exercise regimen.

**Discussion:**

As the elderly population was shown to be less proficient regarding the use of new technologies, the application at hand was explicitly made simple. Elements of different PT programs were incorporated and quadriceps strengthening exercises were included. application was composed based on the findings of the reviewed literature and then subsequently modified to incorporate the expert's suggestions. Experts mainly expressed concerns regarding the safety of patients during unsupervised physical therapy as well as the safety of the recorded data. Thus, password protection and a split between the physician's and the patient's interface was created.

## Introduction

Multiple studies with varying designs attempted to evaluate telerehabilitation for a multitude of conditions. In the domain of orthopedic surgery, reviews predominantly investigated eHealth-based rehabilitative measures after total hip or total knee arthroplasty. While some authors argued that strong evidence supports the efficacy of telerehabilitative measures after surgery (1), others only found a significant positive effect on one parameter, namely functionality (2). Some reviews concluded that moderate quality evidence supports the use of telerehabilitation to improve pain, while low quality evidence supports functional mobility (3).

A vast number of mobile health applications targeting a variety of diseases and patient populations have been developed in the last decade. Primarily, the development of these applications was driven by financial and economic interest rather than for research and investigative purposes (4). Except for some moderate quality evidence supporting the use of applications that target asthma patients, attendance rates and smoking cessation, mobile applications have not shown any evident impact on the treatment of health conditions. Mixed results and the lack of long-term outcomes have further complicated the issue at hand (5). In the domain of orthopedic surgery, one systematic review examined mobile applications targeting the subset of patients undergoing rehabilitation after total hip or a total knee replacement (6). This review made use of the Mobile Application Rating Scale (MARS), which is reliable and valid instrument in the assessment mobile health application quality (7). The system usability score was also implicated in this review (8). Furthermore, the authors of this study give recommendations for the design of future mobile health applications.

Since osteoarthritis is a disease of old age (9) and since most total knee replacement procedures are performed on the elderly population, the target audience for the application is somehow well defined. However, this proves challenging for the design of a mobile application. Caprani et al. categorize these problems in the following categories: perceptual, psychomotor, cognitive, and physical (10). In addition, Kurse et al. reported that the aging population is facing some main barriers when it comes to the use of mobile health applications. These barriers were, however, described as “great opportunities” for the conduction of future randomized controlled trials (11).

The use of the evidence-based research approach implies that previous similar studies are needed to justify a new study as well as inform its design (12). Confronted with the obvious challenges and barriers affecting application-based physiotherapy by the elderly, the researchers decided that the creation of a suitable application and its implementation into a randomized controlled trial will be a valid and valuable use of research resources.

The aim of this study was to develop a mobile application that enhances the adherence of patients to their physiotherapeutic exercise regimen in the early postoperative rehabilitation period after total knee replacement. This application will be used in a RCT that targets the patient's adherence to rehabilitative measures in the early postoperative rehabilitation phase after total knee replacement. To achieve these goals, the study will mainly orient itself on the previously mentioned work of Bahadori, Wainwright and Ahmed (2018).

## 2 Materials and methods

**Figure 1:**
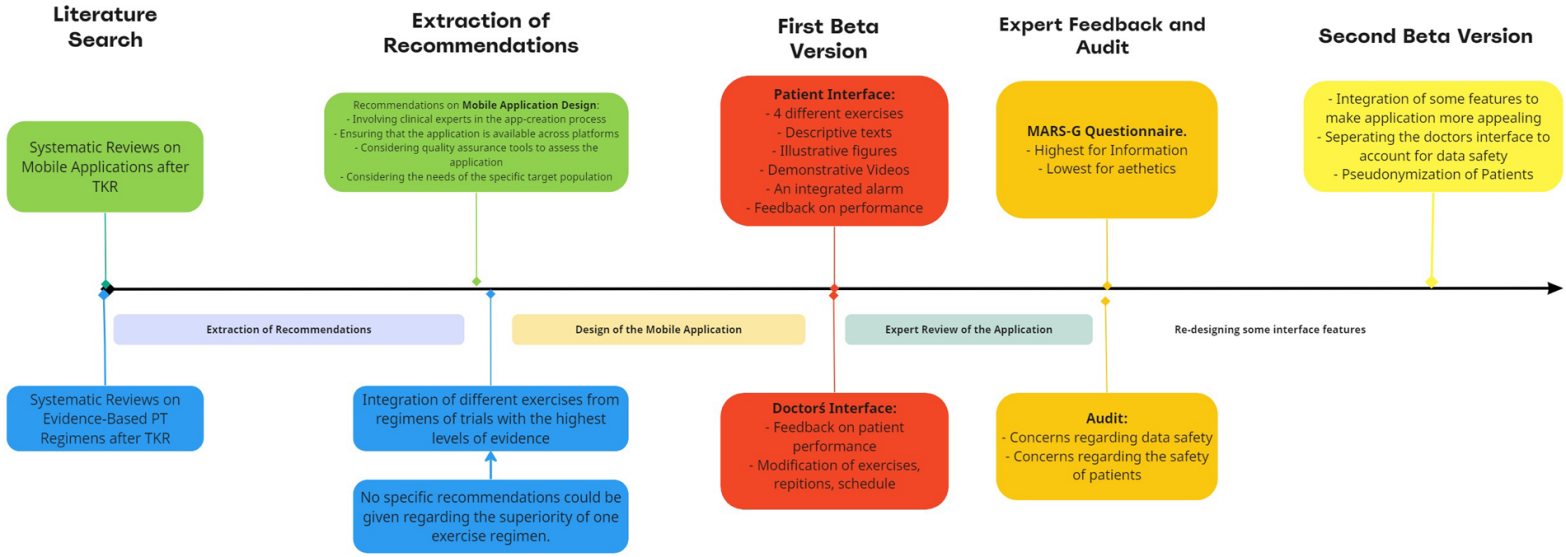
Timeline representing the different steps involved in the conceptualization of the mobile application.

A detailed timeline with the main steps involved in the conceptualization of the application is represented in Figure 1 below.

### 2.1 Literature review

A literature review was conducted to guide the development of the application at hand. One systematic review analyzed multiple commercially available PT-applications designed for TKR patients. The recommendations suggested in the meta-analysis form the cornerstone on which the application is based and are summarized below (6). Furthermore, a literature search aimed at identifying the best early postoperative physiotherapeutic program was conducted.

#### 2.1.1 Recommendations for mobile application design

##### 2.1.1.1 Involving clinical experts in the app creation process

Two physical therapists, two orthopedic surgeons, two software engineers and two health care researchers set out to design an application that meets the needs of the target population (13). Main suggestions included the integration of a simple interface with relatively few bottoms as well as simple exercises that focus on mobilizing the knee joint and strengthening the quadriceps muscle. Furthermore, the inclusion of an explanatory text and descriptive videos was agreed upon.

### 2.2 Ensuring that the application is available across all OS platforms

After reconciliation with the software engineer, it was made clear that the app has the potential to be available on various platforms including IOA and Android.

### 2.3 Considering quality assurance tools to assess the application

After a preliminary version of the application was created, two orthopedic surgeons, two physiotherapists and health care researchers were asked to provide feedback and to rate the application using the German version of the mobile application rating scale (MARS-G) (14). All of these experts were independent from the panel of experts involved in the app creation process. The two orthopedic surgeons from this set will be performing the TKRs in a planned feasibility study. The results of the MARS-G questionnaire and expert suggestions will be presented in the results section. Table 1 summarizes the expertise of the involved research team.

**Table 1:** A representation of the expertise of the research team developing the application.

Experts	Experience
Orthopaedic surgeon 1	- More than 20 years of experience performing TKA. - More than 20 years of experience of knee research - President of the largest society for knee surgery in Europe.
Orthopaedic surgeon 2	- More than 10 years of experience performing TKA. - Implemented the recognition of the University Clinic as an Endoprosthesis Center. - Expert in managing postoperative complications of TKR.
Physiotherapist 1	- More than 10 years of experience regarding rehabilitation after TKR. - More than 10 years of research experience regarding the knee. - Head of research at the University Clinic and Head of the Rehabilitation Committee in the largest European society for knee surgery.
Physiotherapist 2	- Chair of a physical therapy department. - Regular contributions to evidence-based practices in physical therapy.
Health care researcher 1	- Researcher at an affiliated Medical University. - More than 10 years of experience with qualitative research in Rheumatology. - Prior experience in the development and implementation of mobile health applications on various topics including rheumatologic diseases, smoking cessation, and telehealth.
Health care researcher 2	- Researcher affiliated with the medical school. - More than 7 years of experience with qualitative research of the health care system
Software engineer 1	- More than 10 years of experience as a software engineer. - Employed in the private sector at the partnering company.
Software engineer 2	- More than 5 years of experience regarding the development of mobile applications.

### 2.4 Considering the needs of the specific target population

Challenges facing the development of a mobile application for the elderly include cognitive, physical, psychomotor, and perceptual dimensions (10). In most commercially available apps, it is evident that older generations face more challenges regarding mobile application usage. This is evident when considering that most mobile applications are tailored to engage the needs of younger generations (15, 16). However, existing differences can be identified within the age group commonly referred to as “elderly”. That is why it is important to recognize that people of old age born between the years of 1946 and 1964 are more likely to engage with newer technology than people born before 1946 (11). Studies that identify challenges and provide recommendations were previously summarized researchers. In addition to the mentioned recommendations, other studies advice that texts should be placed on the top two-thirds of a bottom in an application targeting the elderly (17).

Other papers discuss that instructions regarding the usage of the apps must be provided. This cannot be done by an instructive paper but should rather be based on a face-to-face interaction. As well, patients must be made aware of the benefits such an intervention will provide (18). Finally, this process of discussing the technology at hand will make older patients confident regarding application usage. This, in turn, will encourage compliance with instructions provided by the application (19).

#### 2.4.1 Evidence-based physical therapy

It is generally agreed upon that quadriceps strengthening should be the main aim of a physiotherapeutic program targeting early postoperative physiotherapy after TKR. The program designed for this application was based on the systematic review (20). A combination of all four mentioned training regimes was chosen to be integrated into the training program. The physiotherapeutic program will include drop and dangle, modified quadriceps setting and squats, each to be executed for ten repetitions. A 50 m walking unit was also integrated into the exercise regimen. Each exercise is to be performed at different times throughout the day until the patients are discharged from the clinic.

The application has an integrated alarm. When the alarm goes off, patients can either snooze it or open the application that directly leads them to the required exercise. If participants decide to snooze the alarm, another one will be set half an hour later and then one hour later. Videos depicting the execution of an exercise unit will be displayed as soon as the patient opens the application upon responding to the alarm. The videos will also be made available in the library of the applications.

After developing the application, based on the previous literature recommendations, the end-user's perspective was obtained. The expert group was composed of two orthopedic surgeons, two physiotherapists and two software engineers. All participants were handed the German version of mobile application rating scale (G-MARS) and were asked to provide suggestions. Findings were thereafter evaluated by the research group and passed on to the app-development team for modification.

#### 2.4.2 Outcome monitoring

**Figure 2:**
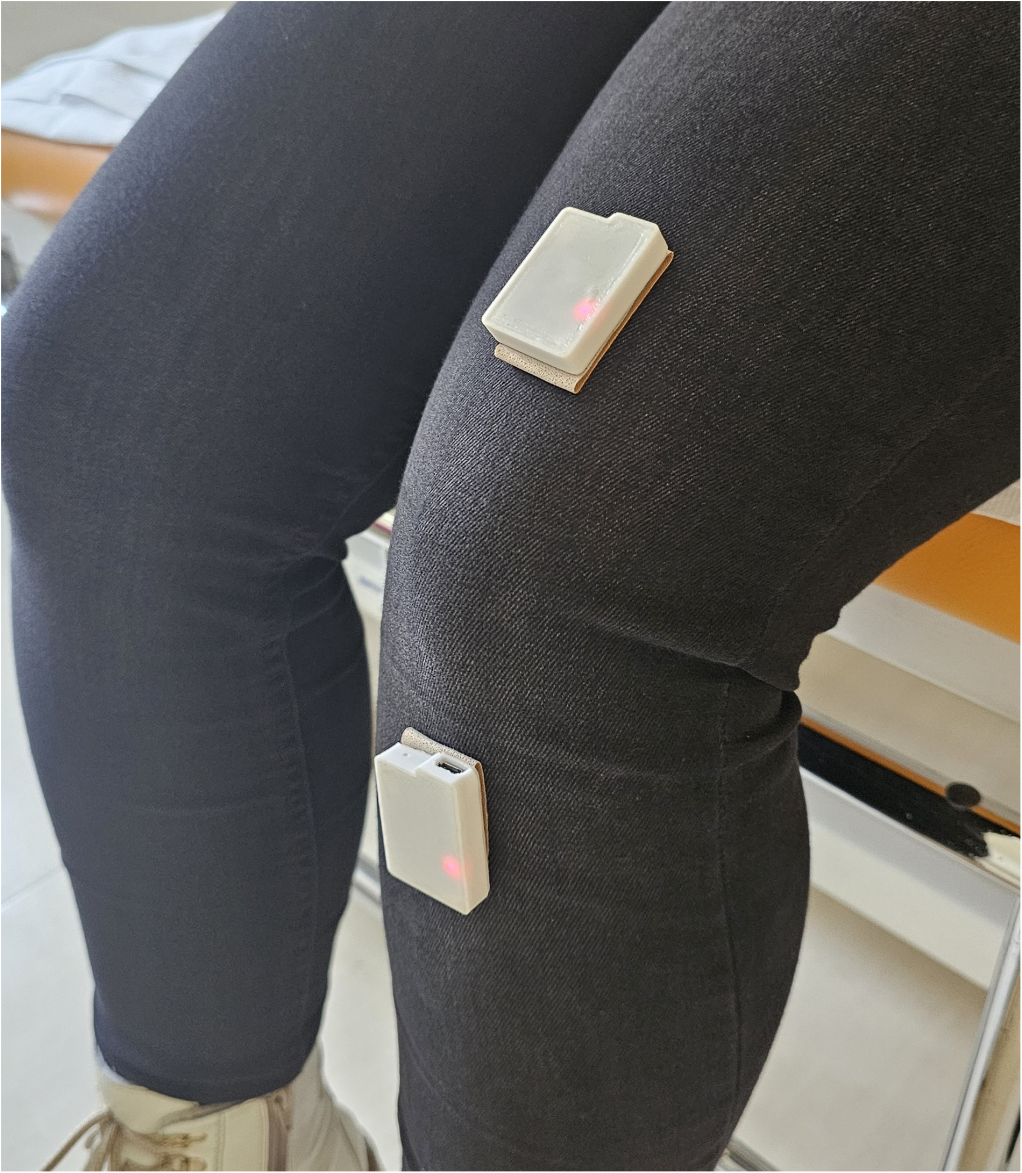
Sensors applied on the distal aspect of the thigh and proximal aspect of the lower leg.

**Figure 3:**
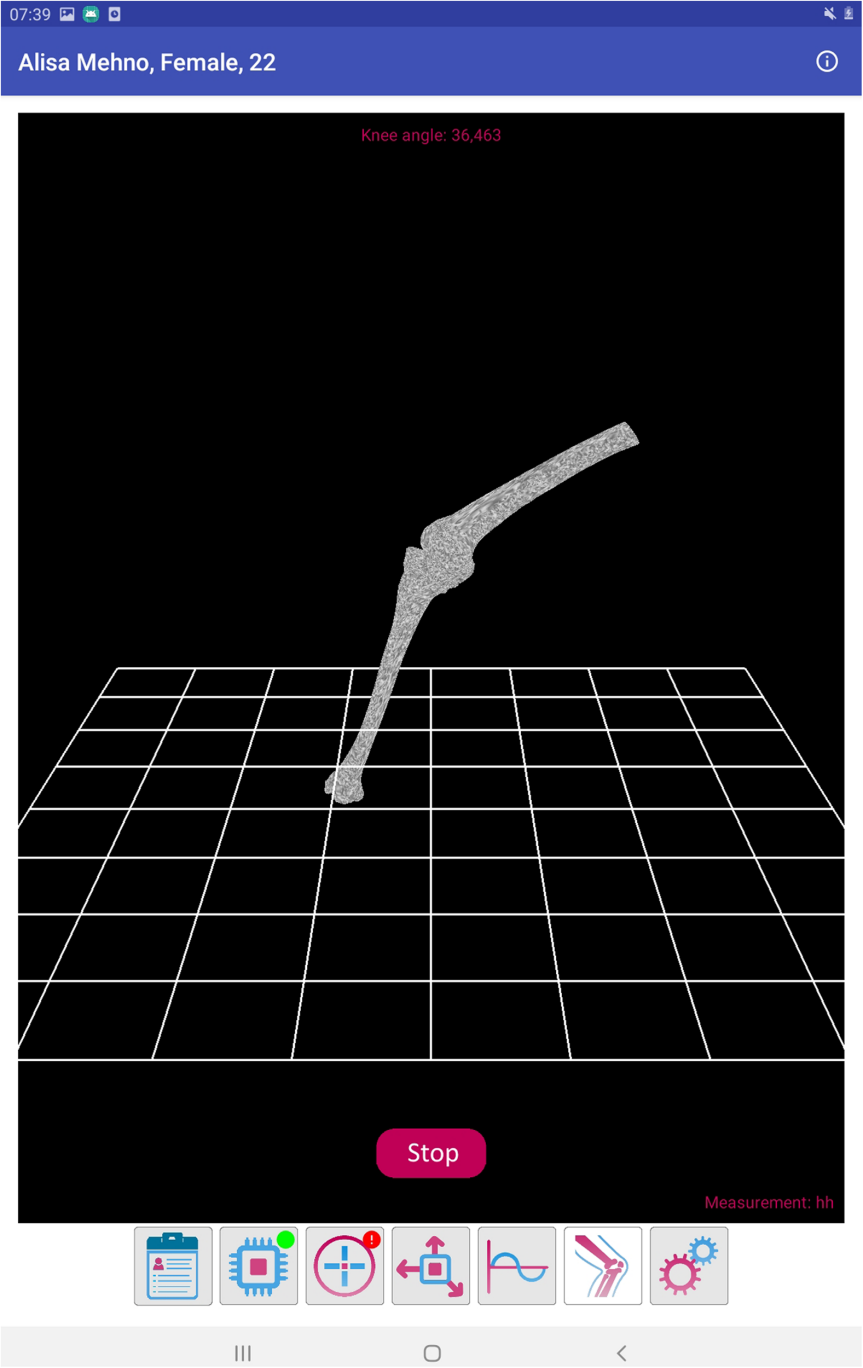
Screenshot detailing the position of the knee joint in the three-dimensional plane.

**Figure 4:**
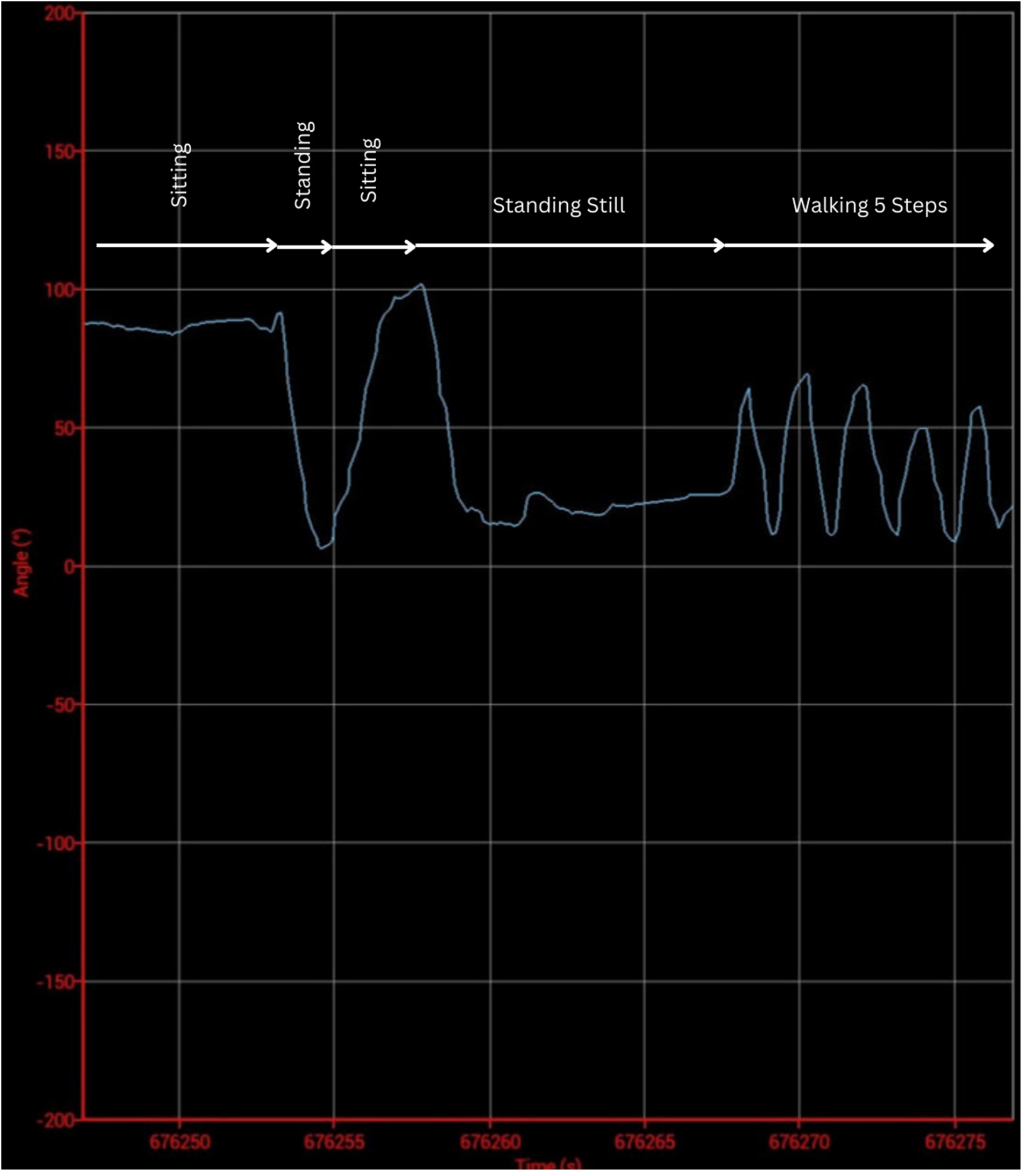
Graphic representation of knee activity in the three-dimensional plane.

As the purpose of the mobile application was to increase the adherence to physical rehabilitation measures, the main outcome of interest was the patient's compliance. Two priorly developed questionnaires were therefore integrated into the mobile application (21, 22). Additionally, the physician's interface was designed to allow communicate with 2 sensors placed on the distal aspect of the upper thigh and on the proximal aspect of the lower leg. Each of these sensors contains three accelerometers and allows for an accurate measurement of the knee position in a three-dimensional plane. As prior studies base the assessment of compliance to physical therapy on questioning patients, only subjective data was reported in the literature till date. The novelty of this application is that it allows to objectively verify the compliance to physical rehabilitative measures. The accuracy and feasibility of these sensors were the subject of a prior study (23). Finally, the wearable sensors are not necessarily required for the functioning of the application. Screenshots from the physician's interface detailing measurements made by these sensors is presented in Figures 2–4 below.

Additionally, validated patient-related outcome measures were integrated into the application.

## 3 Results

### 3.1 Results of the literature review

#### 3.1.1 Recommendations for mobile application design

According to the concept of evidence-based research, new studies and new study methodologies should be informed by the best existing literature. The primary literature search focused on finding an appropriate meta-analysis to inform the design of the mobile application. One systematic review analyzing eHealth applications that guide physical therapy after TKR was found. Recommendations informing the development of new applications were extracted. The methodology section of this paper best describes how the conceptualization of the application at hand adheres to these recommendations.

### 3.2 Specific needs of the elderly generation

As priorly discussed, the target audience for the application at hand is the elderly population. One study systematically identified main challenges of mobile application use by the elderly and presented solutions to these problems. Table 2 was extracted and presents the main challenges and proposed solutions (24).

**Table 2:** A representation of challenges and proposed guidelines identified in the literature review.

Challenges		Proposed guidelines	Comments for our study
Physical	Visual impairment	Large font size	The font size corresponds to 18 in a word document/App provided on a tablet
		High color contrast	The most frequently used color is black to enhance the contrast to the white background
		Avoid blue and green tones	Avoided
	Haptic deterioration	Large button size and spacing	Large Bottoms were used and spacing between bottoms was enhanced
		Minimize the use of keyboard	Keyboard only used by physician to enter the participant's data
		Consider using drag and pinch gestures rather than taps	The app responds to minimal contact with the participants fingers
		Provide audio tactile feedback	Not provided
	Reduced hearing	Provide button to adjust volume	This was not necessary since no auditive feedback was provided
Cognitive	Memory	Intuitive design, avoid the need to recall	The participants can easily find all mentioned features
		Use simple wordings that suit the elderly's semantic field	Simple wordings regarding exercise description were implied
	Speed	Provide ample time to read information	Participants can take as much time as needed to read the available information
		Provide fewer options to choose from so that it is easier to remember the menu path	Participants only have the capacity to choose from a set of options
	Coordination of mental activities	One-level navigation instead of using menu structures	Provided
		Avoid pop-up or multiple overlapping windows	Avoided
		Constant feedback	Not applicable since this will be part of a study to test patient's compliance

### 3.3 Evidence-based PT regimen for early post-operative rehabilitation

Generally, physical rehabilitation after total knee arthroplasty is based on quadriceps and hamstring muscle strengthening. No specific meta-analysis describing the superiority of one exercise regimen over another was found in the literature. One meta-analysis included high quality randomized controlled trials. Although combining the results of the included trials at hand was not possible, all included studies reported superior outcomes. Hence, multiple exercises included from all included were integrated in the training regimen. A detailed description is presented below:

1. Bodyweight squats: In a standing position, the patient grabs onto a stable surface and drops his body while carefully bending the knee and the hip. This exercise is performed to an angle that does not exceed the patient's comfort. The patients then try to return to a standing position whilst mainly focusing on quadriceps activation. This exercise is to be repeated 10 times in the morning during the early postoperative hospital stay.
2. Modified quadriceps setting: Whilst lying in a supine position on the hospital bed, patients try to bend the knee to a degree that does not exceed the patient's comfort. The patients then try to maximally extend the knee. This ensures co-activation of quadriceps and hamstring muscles. This exercise is to be repeated 20 times at noon.
3. Walking: to ensure a unified walking distance, patients are requested to walk from their room down the hallway and then back to a red line and afterwards back to their room. The red duct tape will be placed in a manner that allows to specifically target 50 m. This exercise will be performed in the afternoon.
4. Drop and dangle: This exercise is performed with patients sitting on the hospital bed with the knee bent in a 90-degree angle. The patients try to extend the leg targeting 180 degrees. The leg remains elevated until fatigue is achieved. The leg is then dropped and allowed to dangle back and forth. As contrasted to squats, this exercise allows to isolate the quadriceps muscle. Authors have postulated that it allows to counteract the wear of the prothesis.

### 3.4 First beta version

#### 3.4.1 The doctor’s interface

**Figure 5:**
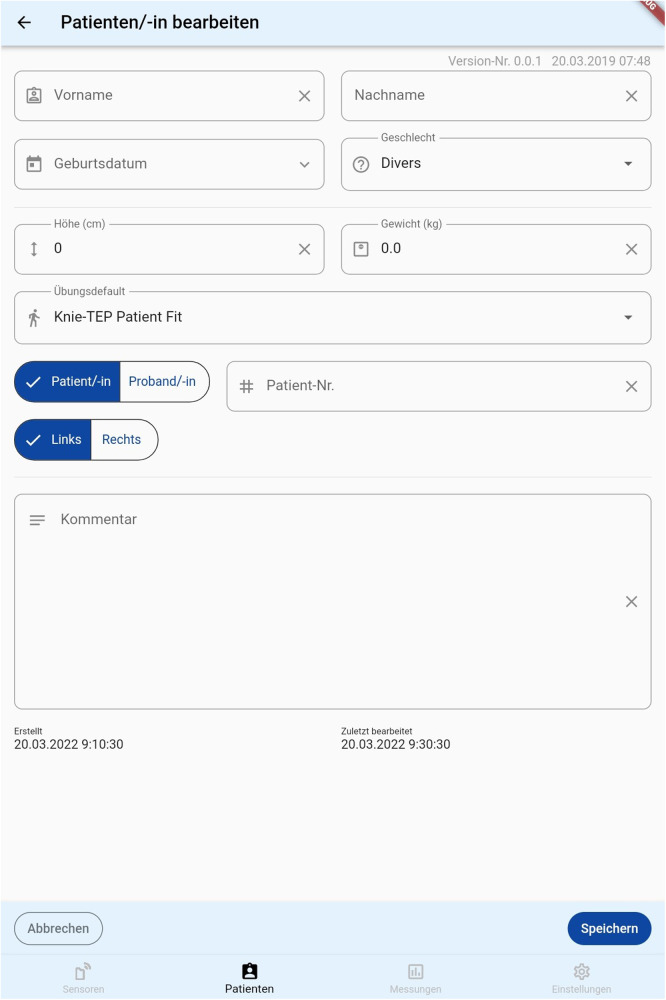
A representation of the initial interface from which a physician can assign a participant.

**Figure 6:**
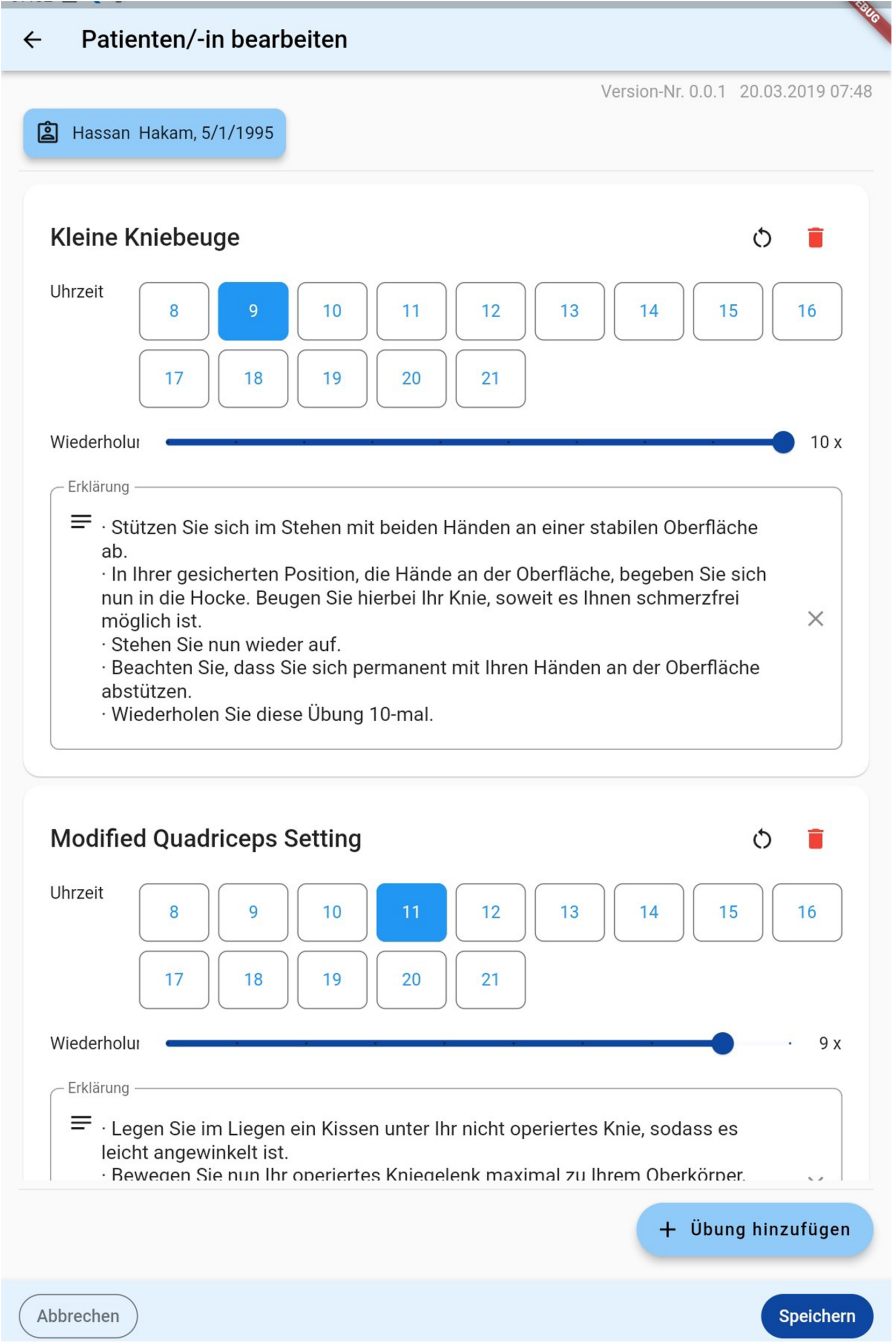
A representation of the “exercise interface”. Doctors can choose when which exercise should be performed and assign the number of repetitions.

The first beta version of the application was then conceived and included the physiciańs interface and the patient's interface. The physician's interface allows the addition of a new participant and the assignment of a pseudonym. Other metadata that can be entered includes age, gender, and BMI. After pseudonymization, only anonymized data is accessible which in turn promotes blinding outcome assessors. This is especially useful in reducing the bias in a subsequent randomized controlled trial. A figure representing the initial assignment of a patient is shown in Figure 5 below. Additionally, the medical doctor can choose to prescribe which exercise should be performed and for how many repetitions. The assignment of exercises is shown in Figure 6 below.

### 3.5 The patient’s interface

**Figure 7:**
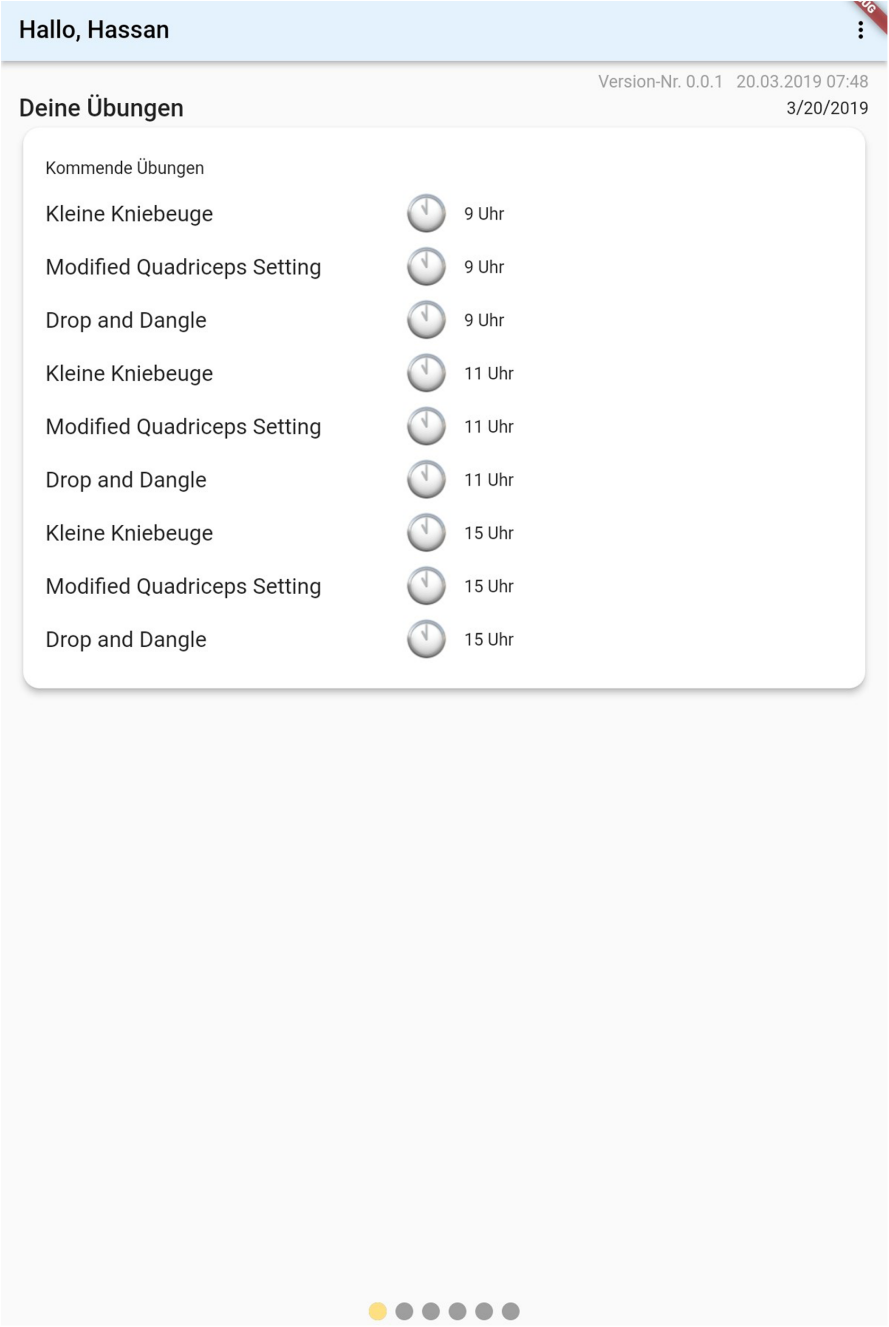
A figure representing the patient's initial interface.

**Figure 8:**
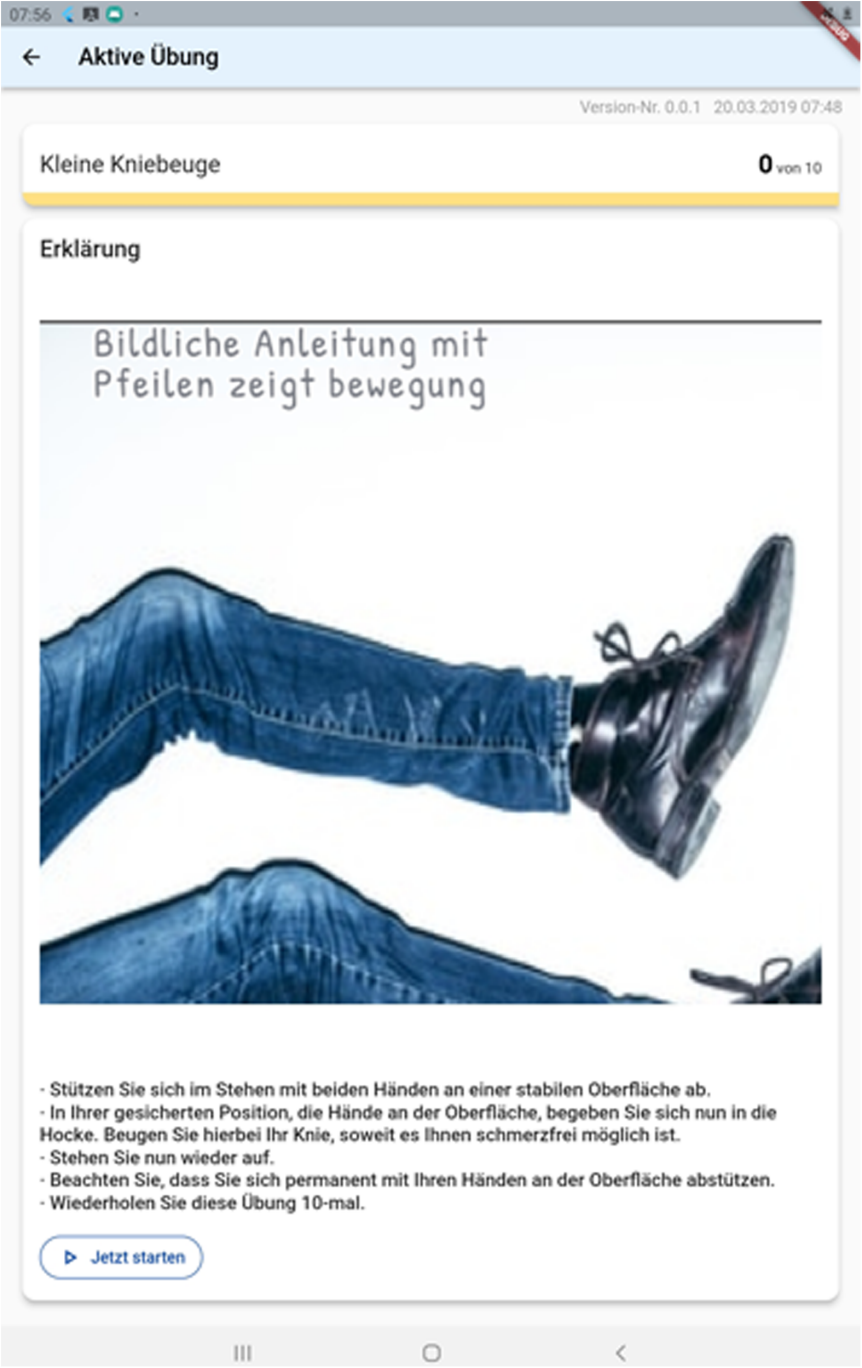
A descriptive text and drawing that is shown upon choosing an exercise.

**Figure 9:**
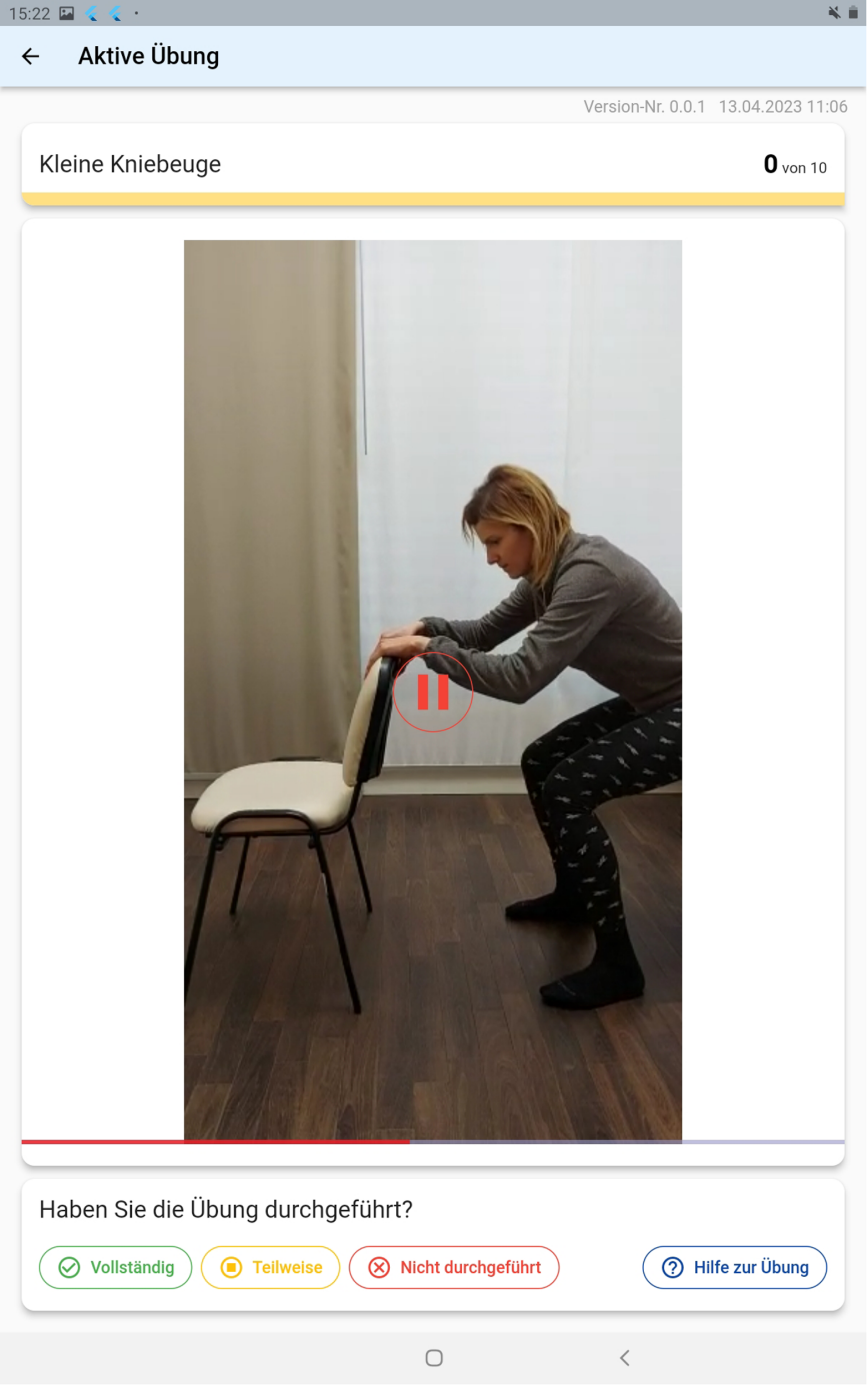
A descriptive video displayed to educate patients on the correct execution of the exercise.

As previously discussed, the interface for patients was explicitly made simple. When opening the application, the prescribed exercises are shown (Figure 5). Upon choosing one exercise, a descriptive text detailing it's execution is displayed. This is shown in Figure 6 below. Patients can then either chose to watch a descriptive video or directly begin to execute the exercises as shown in Figure 6 below. An integrated alarm directly opens the interface shown in Figure 7. Figure 8 represents the textual description of the exercise. Figure 9 is a screenshot of the video interface displaying the correct execution of the exercise.

Additionally, the exercises were dispersed throughout the day. An alarm reminds patients to perform the required exercises.

### 3.6 Links between the interfaces

Initially, the treating physician and physiotherapist select the appropriate exercises for the subpopulation of patients. The intensity, frequency and timing can be regulated using the application. Additionally, the name, the date of birth and the date of the operation are entered. After logging out of the interface, the patient's interface is revealed, the tablet device is handed over to patients and the sensors are fixed onto the patient's knee joint. Answered questions and collected ergonometric data are then transferred to analyzing software. This software can accurately reconstruct the patient's motion in a three-dimensional plane. Stakeholders with an access to the software can then assess the level of activity of individual patients, the range of motion and hence the correct execution of the exercises, the number of repetitions and the overall compliance of patients using the application.

### 3.7 Results of the expert review of the first beta version

#### 3.7.1 MARS-G questionnaire

The results of the MARS-G questionnaire are presented in Table 3 below.

**Table 3:** Average scores from different expert raters for different elements of the MARS-G questionnaire.

Section of the MARS-G	Senior orthopaedic surgeons (2)	Engineers (2)	Physiotherapists (2)	Qualitative (Health care) researchers (2)
A: Engagement	4,20	3.66	3,25	3,20
B: Functionality	4,125	3,75	3,75	4,00
C: Aethetics	3,335	3,6	3,67	2,33
D: Information	4,07	4,14	3,43	3,60
PT: Psychotherapy	4,00	4,25	3,75	4,25
Average	3,945	3,88	3,57	3,48
E: Subjectiv quality	3,75	3,75	3,50	3,00

All the results are expressed as an average of the involved parameter. 1 is the lowest possible score and 5 is highest possible score for an element.

#### 3.7.2 Results of the auditing

Expert auditing regarding the first beta version of the application were as follows:

##### 3.7.2.1 Software engineers

“Data security is a main issue. As the patient's personal information such as name, date of birth, weight, height, and medical condition are saved on the application, one can easily retrieve patient related data. This would present a big privacy issue and should be dealt with. Otherwise, the application does not provide a barrier between the interface the physicians use and the interface the patients deal with. Since the physician can control the number, timing and type of exercise as well as the number of repetitions, it is strongly suggested to create a clear barrier between the two”.

##### 3.7.2.2 Qualitative research experts

“The idea that an app-based intervention might increase the compliance of patients with their physiotherapeutic exercises and thereby their outcome is a relatively good one. The Application at hand seems simple enough for the target population to be used. However, the patients should not be able to interact with the interface that specifies exercises to be executed. Create a barrier between the two interfaces and you are ready to go”.

##### 3.7.2.3 Physiotherapy experts

“The included exercises target the strengthening of the quadriceps as well as knee mobility. Squats seem to be a relatively good exercise, but we would suggest that patients, especially in the first days of rehabilitation, are to be provided with solid support while executing the exercises. Suggestions would include to grab a piece of solid furniture while doing the squats”.

##### 3.7.2.4 Orthopedic surgeons

“The prosthesis will support all the types of mentioned exercises. Caution, however, should be taken to prevent falls during exercise completion to prevent peri-prosthetic fractures”.

#### 3.7.3 Solutions to the presented problems

The following solutions were presented to tackle the before-mentioned issues:

1. Provide a barrier between the physician's and the patient's interface.
2. Provide the application with an automated process that sends the data to the main server and then initiates the deletion of all patient's relevant information after discharge.
3. Include a statement of caution in the application.

### 3.8 Second beta version

The second beta version of the application included all the previously mentioned solutions. The resulting version is presented in Supplementary Material S1 of the manuscript. A detailed description of the functioning is presented in English.

## 4 Discussion

The primary result of this study was informing the design an evidence-based mobile application. The design and content of the application were based on the most recent recommendations found in the academic literature. Recommendations were initially drawn from a systematic review analyzing the effect of specific postoperative rehabilitation exercises on outcomes after knee arthroplasty. Additionally, the challenge of mobile application use by an elderly population was found to be a heavily debated topic in the scientific literature. Solutions to these challenges were extracted and integrated into the design of the application at hand. After expert review of this first version, a second beta version was created to account for the expert's feedback. This version will be used as an intervention in a future randomized controlled trial (RCT). To our knowledge, no known physical therapy application is based on the best available evidence followed by rigorous expert review. Final modifications will be undertaken after patient feedback from the RCT.

As population aging is increasingly placing a toll on healthcare sectors worldwide, innovative solutions have to be found to provide a larger scale delivery of medical interventions with a minimal use of human resources (25, 26). The domains od orthopedic surgery and musculoskeletal rehabilitation are most affected by population aging as osteoarthritis is one of the most prevalent diseases of older age (27, 28). Telerehabilitation has been proven to provide an adequate solution to the many challenges faced by the health care sector (29, 30). As these technologies only require a limited initial effort during conception, end-users including the target populations as well as health care providers profit from their implementation into daily clinical practice. In the domain of musculoskeletal physical therapy, telerehabilitation has been proven to be as effective as face-to-face rehabilitation, further underlining the applicability of electronic health-based interventions targeted in this study (31).

In addition to disburdening the health care system, telerehabilitation seems to pose a practical solution to the large number of adverse events encountered in an inpatient setting. A study by Fu et al. that included approximately fifty-eight thousand participants, reported that patients receiving postoperative rehabilitation in specialized care centers directly after hip arthroplasty were more likely to have wound infections, develop urinary tract infections, be readmitted to the hospital, have wound healing problems, and have respiratory complications (32). Consequently, at-home mobile application-based rehabilitation has the potential to decrease the number of the previously mentioned complications.

Similar previous pilot studies gained primary insights into the opportunities electronic health care applications following joint replacement might provide. Rossi et al. found out that telerehabilitative measures might increase the patient´s engagement and facilitate the personalization of care (33). Furthermore, McKeon et al. emphasized the economic benefits of mobile rehabilitative measures whilst preserving patient satisfaction and providing similar outcomes to face-to-face rehabilitation (34). Additionally, Stauber et al. emphasized the positive effect on self-management capabilities (35). Hence, evidence-based mobile rehabilitative applications have the potential to increase the self-reliance of patients, decrease health care costs whilst preserving the patient's satisfaction with the treatment when compared to traditional rehabilitative measures.

Although an extensive literature review was conducted, no study detailing the conception of a mobile health application was found. Many studies describing the effectiveness of mobile PT applications were found. However, these studies did neither describe the application in the warranted details nor did these studies base the design and content on the current state of evidence. To our knowledge, the paper at hand is the first article describing the conception of an evidence-based PT application. As the topic of evidence-based mobile applications in healthcare is still in its infancy, much work must be done to facilitate their integration into daily clinical practice.

Finally, as mobile technologies are getting more integrated into daily clinical practice, caution must be taken regarding the personalization of treatment delivery. A variety of patients suffering from the same disease might require different therapeutic strategies. Hence, standardizing telerehabilitation might pose serious consequences to individual patients. Therefore, a feedback mechanism allowing the constant monitoring of a patient´s individual status and progress should be accounted for in the conception of a mobile health application. The application at hand allows patients to provide feedback on the number of repetitions upon which the physician can modify the exercises and the number of the performed repetitions.

### 4.1 Limitations

The greatest limitation of this application is the language barrier. The application will be conceived for German speaking people. This makes the application of restricted usability. A second limitation is the absence of a clearly superior exercise regimen in the literature. Although it is generally agreed that quadriceps strengthening is a clear goal, the nature, number of repetitions and time of rest between the subsequent training units is not yet agreed upon. Another limitation would be the lack of feedback from the target population. This will, however, be targeted during an upcoming randomized controlled trial. Additionally, the challenges regarding the compliance of the elderly with sensor application and mobile application use might constitute a limitation. However, the application targets early postoperative rehabilitation after TKA and patients will be under close supervision at our department. Finally, the effect of the application on other stakeholders including doctors, physicians and nurses was not assessed. However, the authors of this and similar studies postulate that the increasing reliance on telerehabilitative measures might moderate the intense strain placed on health care resources.

## 5 Conclusions

This study resulted in the conception of a mobile application that specifically deals with physiotherapeutic rehabilitative measures after total knee replacement. Recommendations of the existing literature were collected and implicated in the design of the application. The perspective of the end-users was appraised using specific tools to specifically assess mobile application.

## Data Availability

The raw data supporting the conclusions of this article will be made available by the authors, without undue reservation.

## Supplementary material

Supplementary Material S1 Final version of the application at hand with a step by step description of application functioning.
