# Supplementary material for: Conception of a mobile health application targeting early postoperative physiotherapeutic care after total knee replacement, a qualitative study
Source: Front Surg. 2025 Jan 27;11:1283202. doi: 10.3389/fsurg.2024.1283202 (PMC11808903; doi:10.3389/fsurg.2024.1283202)
Supplement: Supplementary Material S1 — Final version of the application at hand with a step by step description of application functioning. [file Datasheet1.pdf]

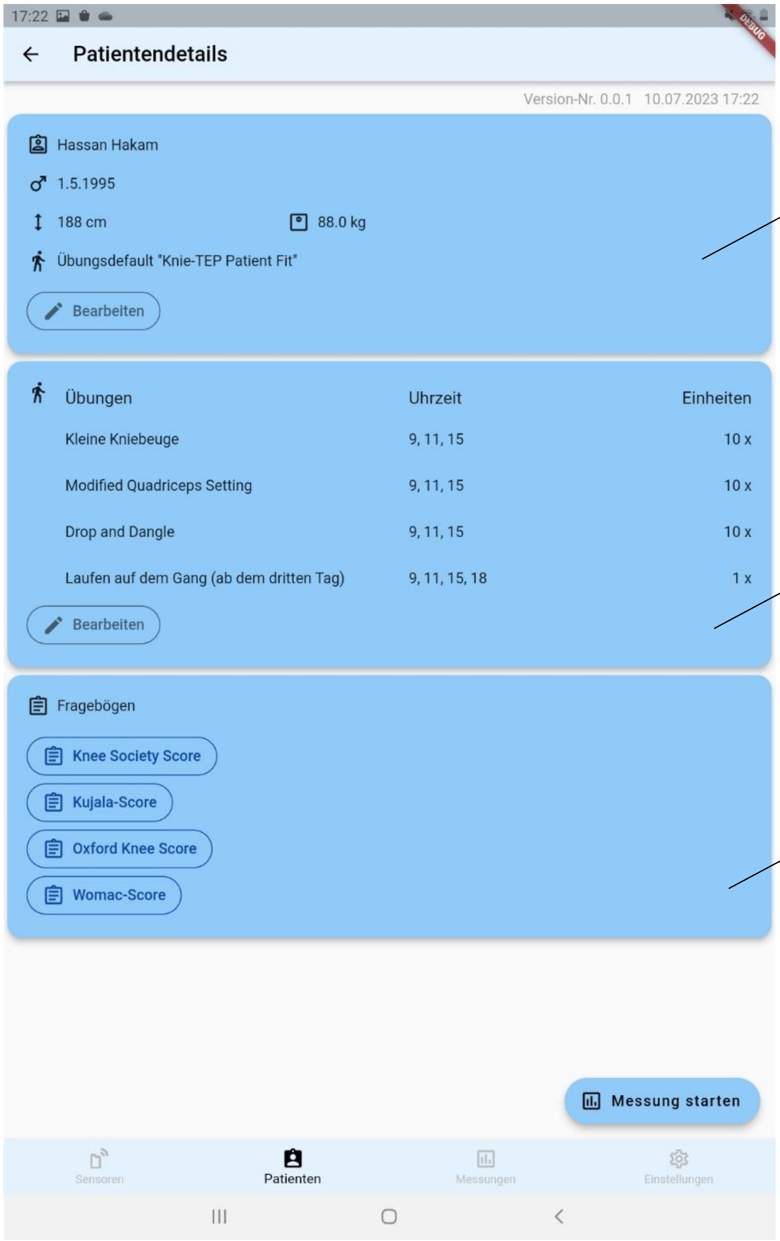

The patient information

The exercises that the patient is required to do. The middle table displays at what time of the day the exercises are to be done and the table to the right displays how many units of each exercise are required.

Forms that the patient is required to fill. Each questionnaire is displayed by clicking on the relevant form.

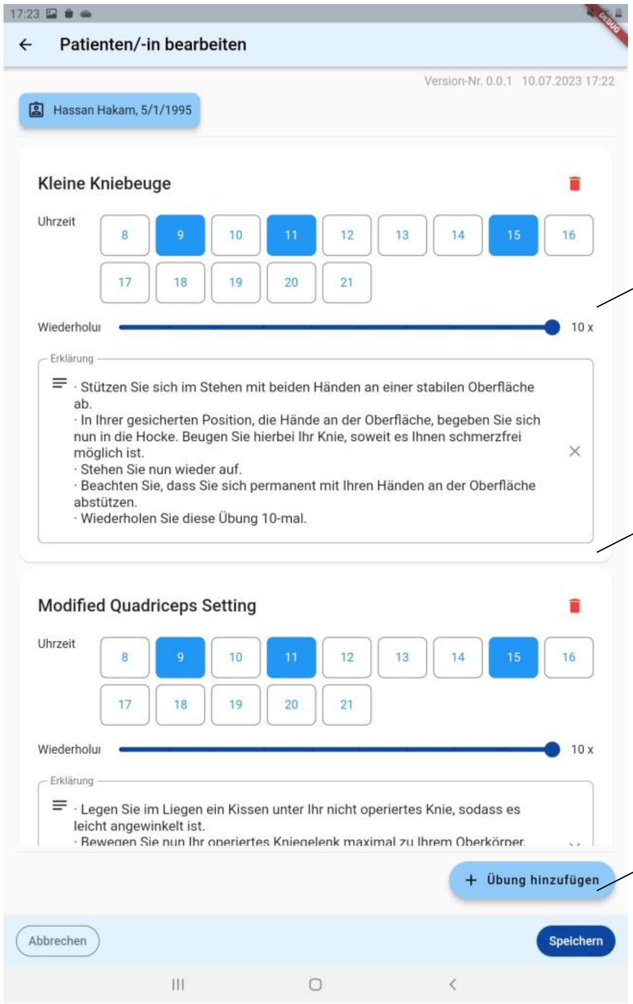

The doctor inserts the type of exercise required to be performed by the patient, at what time of the day and the units to be performed.

In the textbox the doctor gives an explanation of how to exercise should be executed.

By clicking here the doctor can add more exercises.

A schedule of the exercises and time of performance are here displayed.

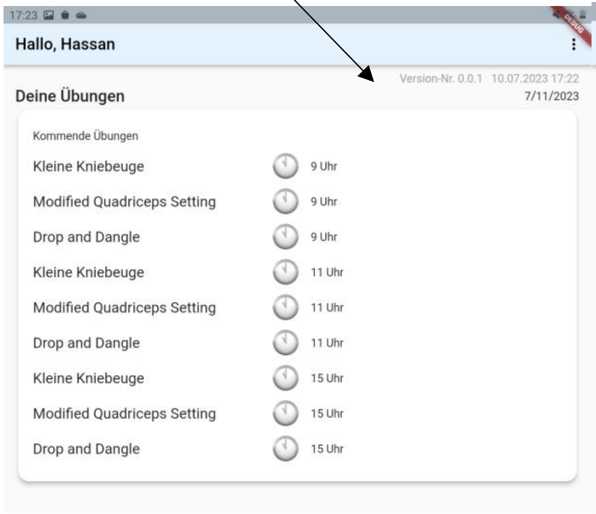

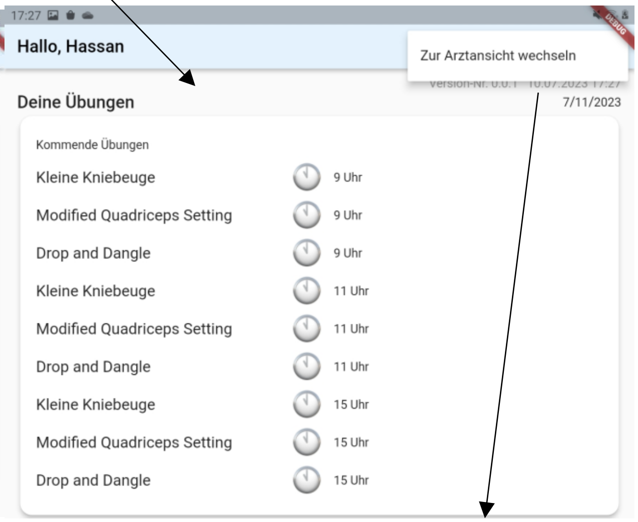

By clicking here you go back to the doctors interphase where a PIN code is required for data protection.

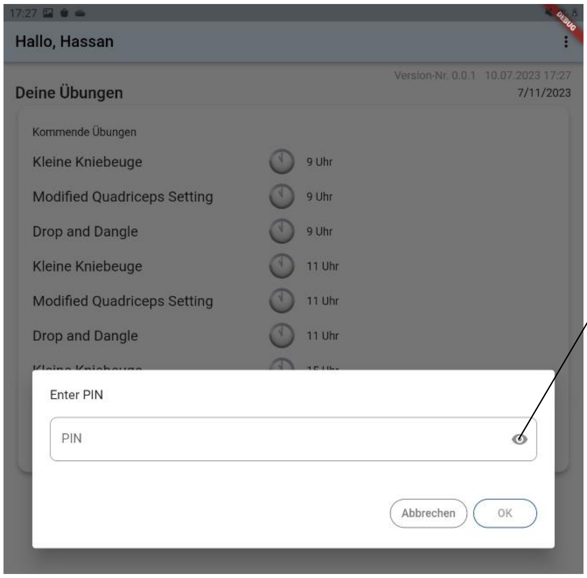

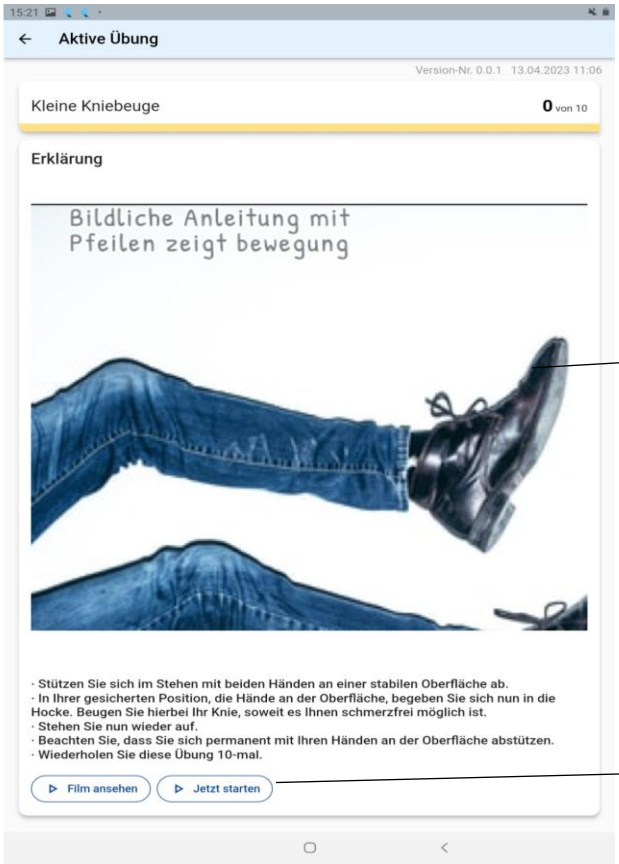

In the following page a pictorial guide is given to show the movement that the patient is asked to perform.
The text below explains how the exercise should be executed and how many units.

By clicking here the patient can chose whether to start immediately the exercise or to watch a short video of how the exercise is performed.

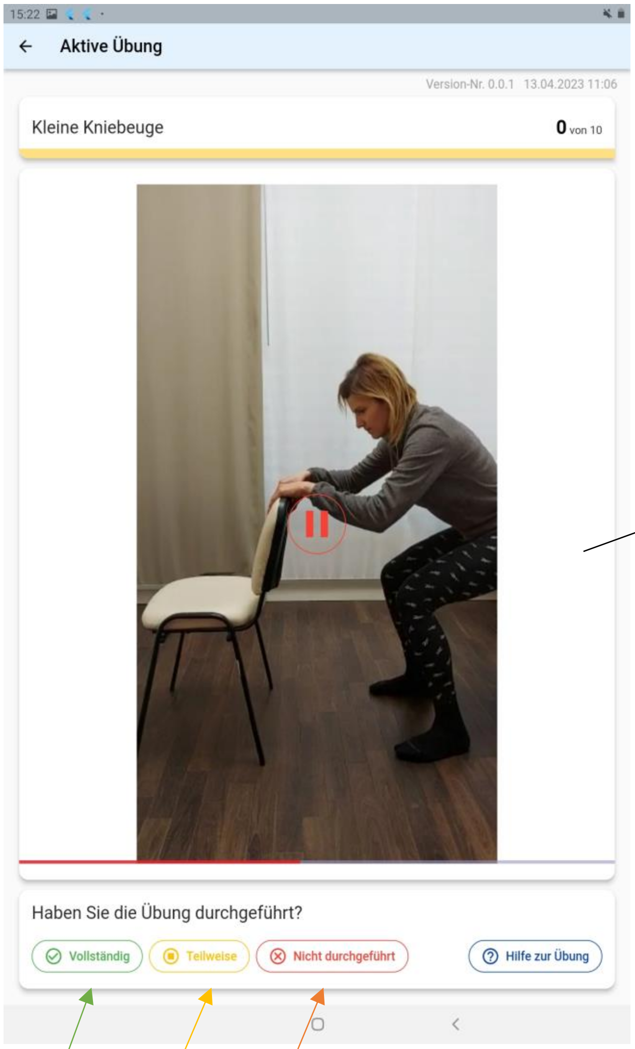

A video that shows how the exercise is executed.

Here the patient clicks if the exercise has been completed, if it has been only partially performed or if the exercise has not been performed.

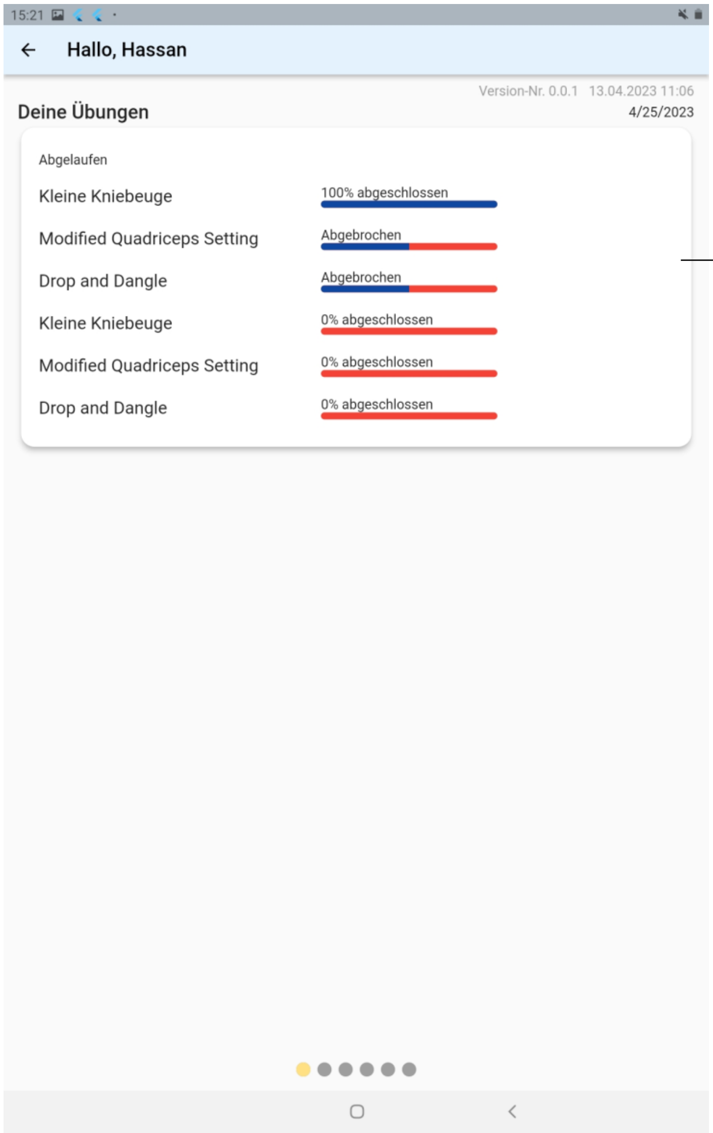

Here the patient can see his/her progress in each relevant exercise .
